# Hydrogen Sensing via Heterolytic H_2_ Activation at Room Temperature by Atomic Layer Deposited Ceria

**DOI:** 10.1002/cssc.202402342

**Published:** 2025-02-03

**Authors:** Carlos Morales, Rudi Tschammer, Emilia Pożarowska, Julia Kosto, Ignacio J. Villar‐Garcia, Virginia Pérez‐Dieste, Marco Favaro, David E. Starr, Paulina Kapuścik, Michał Mazur, Damian Wojcieszak, Jarosław Domaradzki, Carlos Alvarado, Christian Wenger, Karsten Henkel, Jan Ingo Flege

**Affiliations:** ^1^ Applied Physics and Semiconductor Spectroscopy Brandenburg University of Technology Cottbus-Senftenberg Konrad-Zuse-Straße 1 03046 Cottbus Germany; ^2^ ALBA Synchrotron Light Source Carrer de la Llum 2–26 08290 Barcelona Cerdanyola del Vallès Spain; ^3^ Departamento de Química, Facultad de Farmacia Universidad San Pablo CEU Pl. Montepríncipe s/n 28668 Alcorcón Madrid; ^4^ Institute for Solar Fuels Helmholtz Zentrum Berlin für Materialien und Energie GmbH 14109 Berlin Germany; ^5^ Faculty of Electronics, Photonics and Microsystems Wroclaw University of Science and Technology Janiszewskiego 11/17 50-372 Wroclaw Poland; ^6^ IHP – Leibniz-Institut für innovative Mikroelektronik Im Technologiepark 25 15236 Frankfurt (Oder) Germany; ^7^ Faculty of Mathematics and Physics, Department of Surface and Plasma Science, V Holešovičkách 2 Present address: Charles University Prague 18000 Czech Republic

**Keywords:** ALD, ceria, NAP-XPS, reduction, room temperature

## Abstract

Ultrathin atomic layer deposited ceria films (<20 nm) are capable of H_2_ heterolytic activation at room temperature, undergoing a significant reduction regardless of the absolute pressure, as measured under *in‐situ* conditions by near ambient pressure X‐ray photoelectron spectroscopy. ALD‐ceria can gradually reduce as a function of H_2_ concentration under H_2_/O_2_ environments, especially for diluted mixtures below 10 %. At room temperature, this reduction is limited to the surface region, where the hydroxylation of the ceria surface induces a charge transfer towards the ceria matrix, reducing Ce^4+^ cations to Ce^3+^. Thus, ALD‐ceria replicates the expected sensing mechanism of metal oxides at low temperatures without using any noble metal decorating the oxide surface to enhance H_2_ dissociation. The intrinsic defects of the ALD deposit seem to play a crucial role since the post‐annealing process capable of healing these defects leads to decreased film reactivity. The sensing behavior was successfully demonstrated in sensor test structures by resistance changes towards low concentrations of H_2_ at low operating temperatures without using noble metals. These promising results call for combining ALD‐ceria with more conductive metal oxides, taking advantage of the charge transfer at the interface and thus modifying the depletion layer formed at the heterojunction.

## Graphical abstract

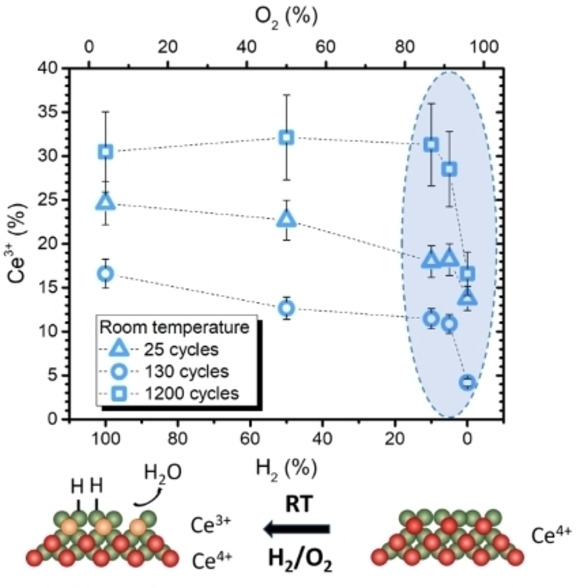

## Introduction

Moving towards a new, renewable energy system based on green energy vectors such as hydrogen (H_2_) needs not only direct energy production and storage systems but also the development of secondary components, such as highly sensitive hydrogen (H_2_) gas sensors integrated into mass devices that operate at ambient conditions in terms of temperature and pressure. While the H_2_‐sensor technology is well‐established for use in industry, where expensive materials, human resources, and maintenance protocols can be relied upon, the rapid development of the so‐called hydrogen economy requires low‐cost, low‐maintenance, easy‐to‐use, accurate, and fast H_2_ sensors for use by untrained individuals on a daily basis.^[1]^ In this context, semiconductors have recently attracted much attention due to the change in their electrical conductivity towards exposure to reducing or oxidizing gases.[^2^, ^3^, ^4^, ^5^] For example, besides the most commonly employed compounds SnO_2_ and ZnO,[^6^, ^7^, ^8^, ^9^, ^10^] many other metal oxides such as TiO_2_, Nb_2_O_5_, In_2_O_3_, Fe_2_O_3_, NiO, Ga_2_O_3_, Sb_2_O_5_, MoO_3_, V_2_O_5_, and WO_3_ have shown distinct variations in the electrical resistance during hydrogen exposure.^[11]^

Despite the advantages of nanostructured metal oxide thin films in terms of simple fabrication processes and compatibility with integrated circuits, high sensitivity and short response/recovery times usually require elevated temperatures (>250 °C), which results in high power consumption and poor long‐term stability. Furthermore, lowering the operating temperature while increasing response and gas selectivity has proven challenging, requiring approaches that, in most cases, increase the degree of complexity of the systems. On the one hand, the influence of the intrinsic properties of thin films, such as grain size, porosity, crystallographic orientation, or doping, on the sensor performance has been widely investigated. For instance, morphology and nanostructuring of metal oxides have a tremendous impact, as shown by the inversely proportional relationship between particle size and the sensor response,^[12]^ or the increase in response time resulting from gas diffusion through aggregated particles.^[13]^ These systematic observations have led to the design of mesoporous oxide structures with well‐defined porous patterns[^14^, ^15^] and the use of 1D nanostructures such as nanowires, nanorods, or nanotubes to improve sensing characteristics,[^7^, ^16^, ^17^] combining them in hierarchical nanostructures together with other 0D, 1D, and 2D nano‐blocks.^[18]^ On the other hand, the performance of resistance‐based metal oxide sensors is also enhanced via surface decoration with a noble metal such as Au,[^19^, ^20^] Pt,[^21^, ^22^] or Pd,^[23]^ significantly increasing gas selectivity, response and recovery times, and sensor response.^[24]^ These briefly outlined strategies unavoidably lead to higher costs and difficulties in industrial scale‐up processes, especially considering the synthesis control of nanostructures and the scarcity of noble metals. Therefore, developing metal oxide resistive sensors capable of operating at ambient conditions, i. e., room temperature (RT) and atmospheric pressure, is still an open challenge that makes it desirable to combine metal oxides presenting high chemical reactivity towards reducing gases with sensor designs and deposition techniques compatible with industry requirements.

In this context, the catalytic rare‐earth metal oxide ceria (CeO_x_) finds multiple applications in various fields thanks to the facile exchange between Ce^3+^ and Ce^4+^ states towards exposure to reducing or oxidizing conditions, respectively. The low energy formation of oxygen vacancies on the surface and their easy and relatively fast diffusion towards the bulk[^25^, ^26^] make this oxide an excellent candidate for oxygen (O_2_) resistance‐based sensors.[^27^, ^28^] Moreover, ceria has also shown promising sensing properties for other oxidizing and reducing gases, such as NO_2_ ^[29]^ and CO_2_,^[30]^ or xylene (C_8_H_10_),^[31]^ CO,[^32^, ^33^] and H_2_,[^34^, ^35^] respectively. Despite the promising surface chemistry of ceria, a closer look at the H_2_‐sensing case reveals that its performance is limited to combination with other noble metals[^35^, ^36^] or metal oxides such as CuO_x_,^[37]^ In_2_O_3_,^[38]^ and SnO_2_,^[39]^ showing poor properties when used alone.^[37]^ In this regard, the works of K. Suzuki *et al*.^[34]^ and Y. Song *et al*.^[35]^ summarize the state of the art of ceria H_2_‐sensing to a reasonable extent. While cerium oxide easily reduces or oxidizes in high vacuum conditions at elevated temperatures (500 °C) under exposure to small partial pressures of H_2_ and O_2_, respectively, the combination of both gases results in a step‐like sensor response where ceria will abruptly oxidize or reduce depending on the P(O_2_)/P(H_2_) ratio.^[34]^ To our knowledge, this situation has been only possible to overcome by using noble metals such as Pd, allowing sensor operation at room temperature using low doses of H_2_ in air.^[35]^

Therefore, as with other metal oxides, the development of ceria‐based H_2_ gas sensors capable of operating at room conditions and easily integrable to mass devices requires the use of synthesis or deposition techniques that (i) allow tailoring of the intrinsic properties of ceria films and (ii) are adapted to sensor designs with high aspect ratios to guarantee fast gas kinetics and large effective areas, respectively. In this context, atomic layer deposition (ALD) has gained prominence in the materials community in recent decades due to its excellent atomic‐scale thickness control and conformal deposits on complex 3D geometries, such as mesoporous structures or Si‐nanostructured substrates prepared by complementary metal‐oxide semiconductor (CMOS) compatible technology.[^40^, ^41^] Moreover, ALD deposits are generally amorphous, defective, and non‐stoichiometric,[^42^, ^43^, ^44^] resulting in modified materials properties compared to more ordered counterparts,^[45]^ which could potentially enhance the chemoresistive behavior towards H_2_ sensing.

In this work, we present a novel ALD pathway for synthesizing ultrathin ceria films that are active for H_2_ sensing at room temperature. By using near ambient pressure X‐ray photoelectron spectroscopy (NAP‐XPS) at synchrotron facilities, we were able to monitor a significant surface reduction of the deposited ALD‐ceria ultrathin films when exposed to small traces (<10 %) of H_2_ in an O_2_ atmosphere at room temperature. Besides, we were able to evaluate the influence of the deposit thickness on the total amount of reduction to Ce^3+^ states, with thinner films leading to higher Ce^3+^/Ce^4+^ ratios. By comparing as‐deposited and post‐annealed samples of different thicknesses, we could identify the defect‐healing process related to the high substrate temperature during the growth (250 °C) as the cause for this difference in reducibility. The results presented here find applications in multiple fields, such as CO_2_ conversion in catalysis in addition to H_2_ sensing. As a proof of concept, we show gas sensing measurements employing a resistive sensor (i. e., change in electrical resistance) test structure with a 20 nm thick ALD‐ceria layer that is exposed to low concentrations of H_2_ (100–500 ppm) at relatively low temperatures (<200 °C). As shown, ALD‐ceria active films overcome the current temperature limitations of ceria as a sensor material^[34]^ and open the path to developing novel catalytic and active sensor materials based on non‐ordered ceria.

## Experimental Section

The ceria atomic layer deposition was performed in a stainless steel homemade reactor attached to the preparation chamber of an ultra‐high vacuum system coupled with an X‐ray photoelectron spectrometer.^[46]^ The ceria was deposited by thermal ALD using the precursor tetrakis(2,2,6,6‐tetramethyl‐3,5‐heptanedionato)cerium(IV), Ce(thd)_4_, 99.99 % purity, commercially available from EpiValance, in combination with ozone (~7 %, O_3_/O_2_ mixture) generated with an OXP‐30 Ozone Generator from Oxidation Technologies fed with O_2_ (99.9995 %, Air Liquid). The ALD reactor operates in flow‐type mode using N_2_ (99.9999 %, Air Liquid) as the carrier gas at a pressure range of 1–3 mbar, where the individual gas lines for the precursor and the oxidant are controlled by independent mass flow controllers (F‐111B 200, Bronkhorst). The N_2_ flux was set at 60 sccm. In the flow‐type mode, the ALD reactor turbopump is bypassed and directly pumped by a scroll pump during the deposition (base pressure of 8 ⋅ 10^−3^ mbar in these conditions). The electropolished stainless steel bubbler (STREM) containing the Ce(thd)_4_ precursor was heated to 140 °C, while the N_2_ and O_3_ lines were set at 90 °C, and the walls of the ALD reactor at 120 °C. A substrate temperature of 250 °C, which is within the previously reported ALD temperature window,[^47^, ^48^] was accomplished by radiative heating. The ALD recipe consisted of 1s Ce(thd)_4_ pulse followed by 1.5s of N_2_ purging, continued by an O_3_ dose of 2.5 seconds and subsequent 3s of N_2_ purging. The reactor was purged for 40 seconds between the individual cycles by pumping with the scroll pump (<10^−2^ mbar). The gas input was regulated by ALD pneumatic valves (Swagelok) controlled by LabVIEW‐based (2020 SP1) homemade software. The average growth per cycle (GPC) is 0.2±0.05 Å/cycle. Before the deposition, the substrates, 300 nm thick thermal oxide (SiO_2_) on Si (100) wafers from CrysTec, were annealed at 250 °C under UHV to remove the adventitious carbon and checked by X‐ray photoelectron spectroscopy (XPS). More details about the ALD‐ceria films can be found elsewhere.^[49]^ In the text, the name of the samples will be referred to the total number of ALD cycles, as 25‐CeOx, 130‐CeOx, and 1200‐CeOx, for 25 (0.5 nm), 130 (2.6 nm), and 1200 (24 nm) ALD cycles (thickness), respectively.

*In situ* XPS measurements of the as‐deposited ALD‐ceria films were performed with an Omicron EA 125 hemispherical electron analyzer using non‐monochromatized Mg K_α_ radiation to avoid the overlap of the Ce 3d main photoemission region and the Auger MNN signals from Ce. The pass energy was set to 20 eV, yielding an overall spectral resolution of about 1.0 eV. The sample charging was corrected considering the Si 2s peak at 154.6 eV from the SiO_2_ substrate as an internal reference.^[50]^ After removing the ALD‐ceria samples from the UHV‐ALD system, they were kept under low vacuum conditions to minimize their exposure to the atmosphere and adventitious surface contamination. Near ambient pressure X‐ray photoelectron spectroscopy (NAP‐XPS) measurements were performed at the NAPP end‐station of the ALBA synchrotron′s BL24‐CIRCE beamline,^[51]^ equipped with a PHOIBOS 150 NAP analyzer. An overall resolution of 0.2 eV was estimated at a photon energy of 500 eV, pass energy of 10 eV, and 20 μm exit slit aperture. Measurements were performed at different photon energies, ranging from 380 to 1800 eV, to guarantee the same photoelectron kinetic energies for different regions and to vary information depths. The chamber is equipped with a quadrupole mass spectrometer at the second stage of the analyzer differential pumping system to cross‐check atmosphere composition and possible reaction products. Near ambient pressure hard X‐ray photoelectron spectroscopy (NAP‐HAXPES) experiments were performed at the SpAnTeX end‐station^[52]^ installed at the KMC‐1 beamline of the BESSY II synchrotron facility. The end‐station is equipped with a PHOIBOS 150 HV NAP electron spectrometer, with an overall resolution of 0.8 eV at a photon energy of 6077 eV selected with a Si(422) crystal. Measurements were performed at 6 keV, as confirmed by measuring the Au 4f_7/2_ at 84 eV for a gold sample. For NAP‐XPS and NAP‐HAXPES measurements, the binding energy was corrected using the *u′′′* component of the Ce 3d core level at 916.8 eV as an internal reference^[53]^ and cross‐checked with the Fermi edge for each photon energy. Potential beam damage was considered in all situations and could be ruled out in our laboratory XPS measurements. At the synchrotron facilities, beam damage was avoided by moving the confined beam spot to previously unexposed areas of the sample. After subtracting a Shirley‐type background (and removing the X‐ray satellites in the case of the non‐monochromatized Mg K_α_ radiation), the XPS Ce 3d spectra were fitted using a combination of ten peaks following the procedure reported elsewhere,[^53^, ^54^] with four (*v_o_*, *v′*, *u_o_*, *u′*) and six (*v*, *v′′*, *v′′′*, *u*, *u′′*, *u′′′*) components for the Ce^3+^ and Ce^4+^ states, respectively. The fitting curves were modeled by a symmetrical Gaussian‐Lorentzian (G−L) sum, fixing for each component the same full width at half maximum (FWHM) and G−L ratio values for a given experimental setup (our home lab, ALBA, or BESSY), and using a tail modifier to model the asymmetry of *v* and *u* components, as applied by Skála and coworkers.^[55]^ The spectra have been fitted using the XPS Peak software, version 4.1, while the electron inelastic mean free path (IMFP) through the ceria matrix was calculated using the Tanuma, Powell, and Penn formula IMFP‐TPP2M.^[56]^ The CeO_x_/SiO_2_ film characterization was completed *ex situ* before and after the NAP‐XPS experiment by μ‐Raman spectrometry using a Renishaw InVia μ‐Raman spectrometer equipped with a 532 nm wavelength laser.

Gas sensing measurements were performed using a mixture of H_2_ in Ar as the activating agent, subsequently recovered by synthetic air produced by an Atlas Copco GX3 FF compressor with an activated carbon adsorbing filter (Donaldson AK series) and an integrated air drier that guaranteed humidity levels lower than 10 %. Each measurement cycle lasted 20 minutes, with 15 minutes of activation in hydrogen followed by 5 minutes of recovery in air. The gas flow of 1000 sccm was controlled using MKS mass flow controllers. The tested 20 nm thick ALD‐active deposits were grown on BVT ceramic substrates with interdigitated electrodes. The changes in the electrical resistance were measured with a Keithley 6517A electrometer at different temperatures (25–200 °C).

## Results and Discussion

### As Deposited ALD‐CeO_x_ on SiO_2_

**Figure 1:**
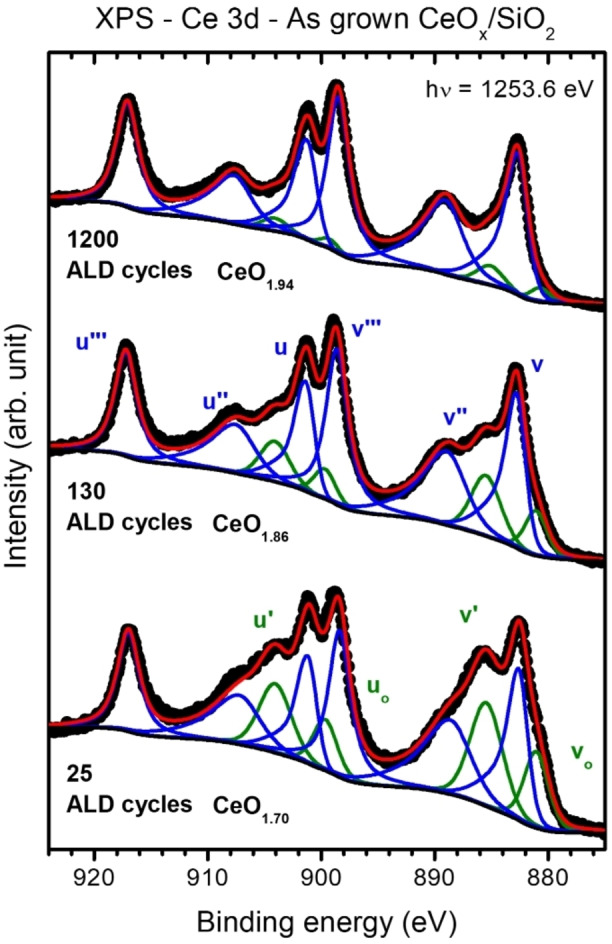
From bottom to top, in‐situ Ce 3d XPS spectra of as‐deposited ALD‐ceria films on SiO_2_ (black circles) with 25 (25‐CeO_x_, ~0.5 nm), 130 (130‐CeO_x_ ~2.6 nm), and 1200 (1200‐CeO_x_ ~24 nm) ALD cycles (thickness), respectively. The red line corresponds to the fit resulting from the Ce 3d deconvolution into ten components, four for Ce_2_O_3_ (in green) and six for CeO_2_ (blue). The measurements were taken using a non‐monochromatized Mg K_α_ source, subtracting the corresponding satellites before the fitting.

As reported in our previous work,^[49]^ the ALD reaction mechanism using Ce(thd)_4_/O_3_ is highly complex, being determined by a change in reaction mechanism when the precursor interacts with the substrate or the atomic layer deposited ceria surface, i. e., the so‐called hetero‐ and homodeposition regimes, respectively.^[57]^ Deposits below ~10 nm grow as micrometric flakes exhibiting a higher amount of Ce^3+^ states that can be related to intrinsic defects within the ALD films, essentially resulting from the interaction with the substrate and, less importantly, from the morphology.[^58^, ^59^] Beyond approximately 500 ALD cycles, these flakes eventually coalesce in a continuous film where the Ce^4+^ states dominate. As it has been shown for ceria films grown on a variety of substrates using other physical vapor deposition techniques,^[60]^ these differences in ceria′s chemical state and structure, combined with a strong interaction with the substrate, might lead to modifications in the reactivity towards exposure to reducing and oxidizing environments.[^61^, ^62^, ^63^] Therefore, to test the role of ALD‐ceria intrinsic defects, three ALD‐ceria deposits of different thicknesses were grown on SiO_2_ substrates in our facilities and subsequently characterized *in‐situ* by XPS. As shown by the fitted Ce 3d XPS spectra in Figure 1, the Ce^3+^/Ce^4+^ ratio of the as‐grown layers depends on the number of ALD cycles, presenting a gradual transition from more reduced ultrathin deposits cycles, i. e., 25 cycles and thickness of ~0.5 nm, to thicker and more oxidized ones for, i. e., 130 and 1200 cycles and 2.6 and 24 nm thick, respectively. As stated in the *Experimental section*, hereafter, samples with 25, 130, and 1200 ALD cycles are called 25‐CeO_x_, 130‐CeO_x_, and 1200‐CeO_x_, respectively.

### Room Temperature Reactivity Towards H_2_ and O_2_ Mixture

**Figure 2:**
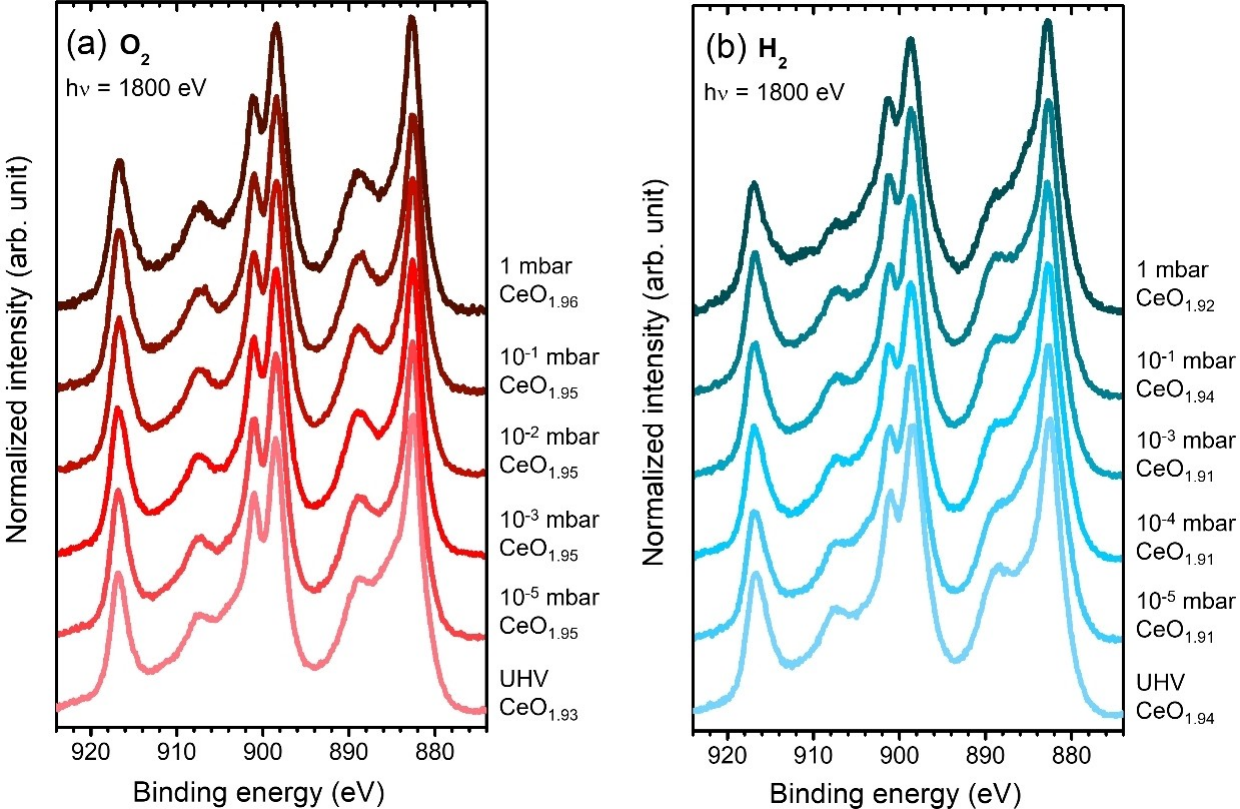
Ce 3d PES spectra of 130‐CeO_x_ (~2.6 nm) sample taken at room temperature under (a) O_2_ and (b) H_2_ atmospheres at different pressures ranging from <10^−8^ mbar (UHV) to 1 mbar, as indicated on the right side. The ceria film composition estimated by fitting the corresponding Ce 3d spectra (CeO_x_, where 1.5<×<2) is given on the right side for each atmosphere and pressure step. The photon energy was set at 1800 eV.

The ALD‐ceria response towards reducing (H_2_) and oxidizing (O_2_) environments was studied under *operando* conditions by near‐ambient pressure XPS. Although ALD‐ceria samples partially oxidize after exposure to ambient conditions during transportation, once introduced in the UHV system (base pressure <10^−8^ mbar), a Ce^3+^/Ce^4+^ ratio closer to the initial state is restored. Figure 2 shows the photoelectron spectroscopy (PES) Ce 3d spectra of the 130‐CeO_x_ sample after exposure to different partial pressures of O_2_ (Figure 2a) and H_2_ (Figure 2b) at room temperature. The ~2.6 nm thick ceria deposits were easily oxidized to approximately CeO_1.95_, completing the transformation quickly without any further changes on the subsequent Ce 3d spectra (each taking roughly 2 minutes). The oxidation state was almost independent of the oxygen partial pressure, P(O_2_), reaching a saturated stage from low 10^−5^ mbar. We observed a similar scenario when the same sample was subsequently exposed to different hydrogen partial pressures, P(H_2_). The sample was immediately reduced to a saturated stage of CeO_1.91_ from low 10^−5^ mbar of pure H_2_ atmosphere (only a slight oxidation at 10^−1^ mbar breaks this tendency, probably an experimental artifact due to the accumulation of water in the chamber as a result of the compromise between H_2_ input flow and pump output at that pressure, as confirmed by quadrupole mass spectroscopy). This behavior was identical for the ultrathin (0.5 nm, 25‐CeO_x_) and thicker (24 nm, 1200‐CeO_x_) samples, which exhibited only slightly different stoichiometries at the saturated states (not shown here). Therefore, ALD‐ceria deposits rapidly and reversibly oxidize (reduce) for very low doses (<1000 Langmuir) and independently of the absolute O_2_ (H_2_) partial pressure at RT, similar to Suzuki and coworkers′ results obtained at 500 °C for magnetron‐sputtered samples.^[34]^ We stress the importance of this result in terms of the future mixing of O_2_ and H_2_ discussed in the following lines: as indicated in the just mentioned work, the response of ceria towards H_2_ and O_2_ mixtures at a fixed temperature will only depend on the ratio of the individual partial pressures of the oxidizing and reducing gases, regardless of the absolute pressure.

**Figure 3:**
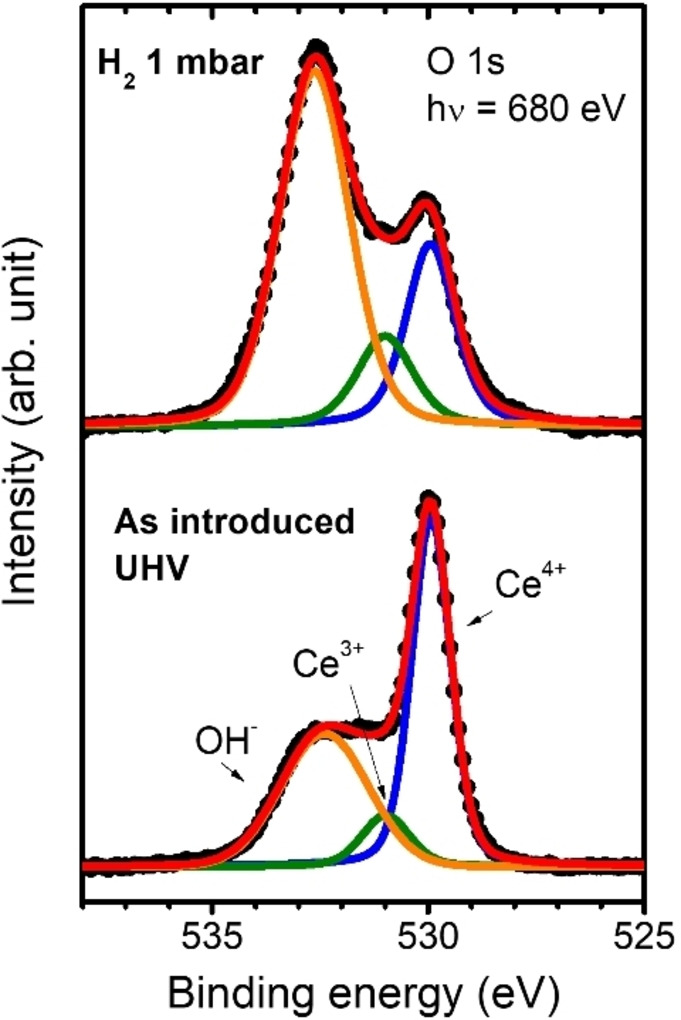
O 1s PES spectra of 1200‐CeO_x_ (~24 nm) sample taken at room temperature as introduced (bottom) and under 1 mbar H_2_ atmosphere (top). The used photon energy (hν=680 eV) provides a photoelectron kinetic energy of approximately 150 eV, ensuring a highly surface‐sensitive measurement.

A closer look reveals that ceria is only slightly reduced (oxidized) from the initial equilibrium state at ultra‐high vacuum conditions. Moreover, the used photon energy of 1800 eV for measuring the spectra in Figure 2 implies an IMFP of about 16 Å, which highlights the surface‐sensitive nature of these measurements; thus, the reduction is limited to the uppermost region of the films. These observations agree with the extensive experimental and theoretical literature describing ceria′s reduction mechanism with H_2_,[^64^, ^65^, ^66^] which can be summarized in a three‐step process (i, ii, iii) leading to two possible scenarios: (1) a hydroxylated surface or (2) the formation of an oxygen vacancy with the release of an H_2_O molecule. In the first scenario, the hydrogen molecule is activated at the ceria surface (i) and reacts with the oxygen of the ceria matrix, creating two hydroxyl species and two Ce^3+^ cations by electron transfer (ii). While the hydroxyl formation marks the endpoint of scenario 1, in the second scenario, an oxygen vacancy is formed through recombination and desorption processes concomitant with the release of water (iii). Using the Kröger‐Vink notation,^[67]^ the scenarios (1) and (2) can be expressed under equilibrium conditions as:
(1)

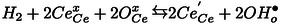



(2)

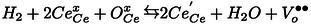




where $Ce_{Ce}^{x}$ and $O_{Ce}^{x}$ represent Ce^4+^ cations and O^2−^ anions in the CeO_2_ matrix, respectively, and 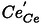 represents Ce^3+^, $OH_{o}^{•}$ the surface hydroxyl species, and $V_{o}^{••}$ an oxygen vacancy. According to Fernández‐Torre *et al*.,^[65]^ the hydrogen is strongly bound after the heterolytic dissociation step (ii, activation barrier ~1 eV), thus preventing it from oxygen vacancy formation at RT. This has been confirmed by analyzing the O 1s spectra, which allows differentiating between the Ce^3+^ states associated with oxygen vacancies or OH^−^ species (in contrast, the Ce 3d spectra would only indicate the total amount of Ce^3+^ independently of their chemical environment). Figure 3 depicts the O 1s spectra of the 1200‐CeO_x_ film taken before and after H_2_ exposure at 1 mbar, showing three peaks at 529.9 eV, 531.0 eV, and 532.5 eV related to Ce^4+^, Ce^3+^ and OH^−^ species, respectively. The Ce^3+^ and OH^−^ related components present a shift to higher binding energies of about 1.1 eV and 2.6 eV to the Ce^4+^ matrix component, respectively, in agreement with the literature.[^68^, ^69^] The broader OH^−^ component of the spectrum taken from the ‘as introduced’ sample is most probably related to an overlap with components from adventitious contamination, as confirmed by the evolution of the C 1s intensity. Therefore, the higher increase of OH^−^ species compared to Ce^3+^ after exposure to H_2_ confirms a predominance of the hydroxylation process (1), as expected at room temperature. Besides, the constricted reduction to the topmost sublayer under these conditions would be additionally related to the relatively high energy barriers of the oxygen vacancy diffusion towards the bulk and the increase of oxygen vacancy energy formation with successive oxygen vacancy formation.^[66]^ In this sense, the obtained composition of approximately CeO_1.92_ matches well with the calculated stoichiometry once the formation of oxygen vacancies saturates the CeO_2_ (111) and (100) surfaces in a crystalline cerium oxide matrix. Although the ALD‐ceria deposit represents a non‐ideal but polycrystalline and defective system with an expected disordered surface, this different atomic structure could lead to an additional entropic stabilization mechanism that reduces the initial energy barriers for steps (i) and (ii), as has been proved theoretically in the case of water adsorption on ceria,^[70]^ thus explaining the relatively facile reduction at such mild conditions compared to other ultrathin deposits of well‐ordered ceria.^[62]^

**Figure 4:**
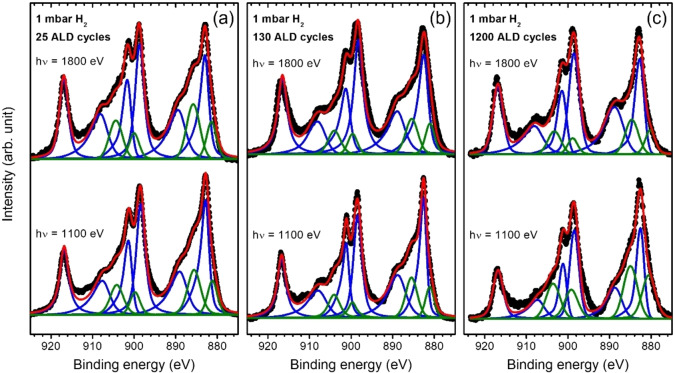
Ce 3d PES spectra of 25‐CeO_x_ (a), 130‐CeO_x_ (b), and 1200‐CeO_x_ (c) samples taken at room temperature under 1 mbar of H_2_ using 1800 and 1100 eV photon energies (black circles, top and bottom, respectively). The fitting (red curves) was performed after background subtraction using the same components as in Figure 1.

Although this discussion indicates that the reversible oxidation/reduction of ALD‐ceria films affects only the surface regions at RT, we observe how this process is strongly influenced by the initial ceria state related to the ALD growth mechanism and the interaction with the substrate. Figure 4 shows the fitted Ce 3d region taken at 1 mbar of H_2_ at two different photon energies, 1100 and 1800 eV, i. e., more and less surface sensitive, respectively, for the three studied samples 25‐CeO_x_ (a), 130‐CeO_x_ (b), and 1200‐CeO_x_ (c). Two effects must be considered when analyzing these results: (1) the initial gradient in the Ce^3+^ states resulting from the ceria/substrate interaction and associated change in the ALD reaction mechanism during the early stages of growth,^[49]^ and (2) the reduction of the surface induced by exposure to a pure H_2_ atmosphere. This way, the ultrathin (25‐CeO_x_) deposit shows a higher concentration of Ce^3+^ states at the film/substrate interface (1800 eV data) than at the surface (1100 eV data), while the thicker deposit is clearly more reduced at the surface, as expected. Therefore, the initial and final state after H_2_ exposure in terms of Ce^3+^ states and its depth distribution will depend on the initial fixation of Ce^3+^ states during the ALD process, which can significantly affect sensing or catalytic properties due to modifications in the depletion layer induced by the charge transfer.[^38^, ^68^] Furthermore, the thickness of the reduced layer can be estimated from the Ce^3+^ and Ce^4+^ intensities extracted from the Ce 3d fittings considering a simple model where a homogeneous, step‐like Ce_2_O_3_/CeO_2_ distribution is assumed:

(3) $$I_{LayerCe^{3+}}\;\;=I_{\infty }^{LayerCe^{3+}}\left[1-e^{\frac{-d}{\lambda_{CeO_{x}}}}\right]$$

(4) $$I_{LayerCe^{4+}}\;\;=I_{\infty }^{LayerCe^{4+}}e^{\frac{-d}{\lambda_{CeO_{x}}}}$$

**Figure 5:**
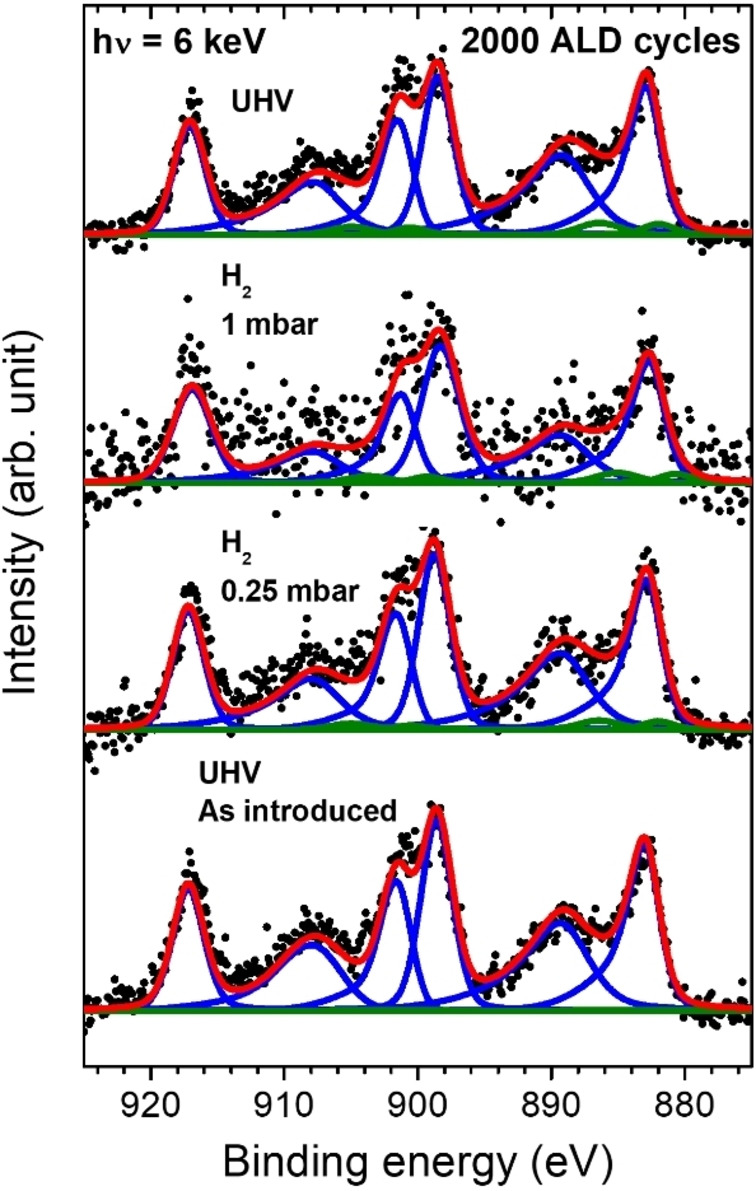
Ce 3d HAXPES spectra (black circles) of an ALD‐ceria film with 2000 cycles taken at room temperature with a photon energy of 6 keV. From bottom to top, the sample was subsequently measured as introduced (UHV conditions, <10^−8^ mbar), at 0.25 and 1 mbar of H_2_, and again under UHV. The fitting (red curves) was performed after background subtraction using the same components as in Figure 1.

Here, $I_{LayerCe^{3+}}$ and $I_{LayerCe^{4+}}$ denote the intensities extracted from the Ce 3d fitting for the Ce^3+^ and Ce^4+^ states, respectively, $I_{\infty }^{LayerCe^{3+}}$ and $I_{\infty }^{LayerCe^{4+}}$ the calculated total intensity of pure Ce^3+^ and Ce^4+^ samples measured under such experimental conditions, $\lambda_{CeO_{x}}$ is the IMFP at the specific photon energy within the ceria matrix (calculated as an average of the IMFPs of CeO_2_ and Ce_2_O_3_ matrix), and *d* is the thickness of the reduced ceria layer. Table 1 summarizes the obtained results, showing a better accordance between the estimated Ce_2_O_3_ thicknesses for different excitation energies when the considered sample is thicker, converging into a value of around 3 Å that approximately corresponds to the reduction of the first atomic layer of ceria (assuming an average value between the Ce−Ce and Ce−O distances in the fcc structure, 3.8 Å, and 2.3 Å, respectively), as previously discussed. These results are confirmed by the NAP‐HAXPES measurements shown in Figure 5. By using a high photon energy of 6 keV, i. e., IMFP of ∼62 Å, about four to ten times the values for photon energies of 1800 and 1100 eV, respectively, we observe that the amount of Ce^3+^ states after RT exposure to H_2_ at different pressures is significantly lower compared to the surface‐sensitive NAP‐XPS measurements (Figure 4), definitely confirming the absence of oxygen vacancies diffusion towards the bulk and the modification of the surface depletion layer due to charge transfer during the hydroxylation step.

**Table 1:** Used values for equations (3) and (4) to calculate the Ce_2_O_3_ thickness considering a simple model that assumes a homogeneous, step‐like Ce_2_O_3_/CeO_2_ distribution. The thickness of the Ce_2_O_3_ (CeO_1.5_) layer is also provided.

Sample	hν (eV)	λ (Å)	I [Ce^3+^] (cps)	I [Ce^4+^] (cps)	at % (Ce^3+^)	at % (Ce^4+^)	CeO_1.5_ layer thickness (Å)
**25 ALD cycles**	1100	6.07	191053	747322	20.4	79.6	1.5
	1800	16.02	20554	77870	20.9	79.1	4.1
**130 ALD cycles**	1100	6.07	69160	70152	18.1	81.9	1.3
	1800	16.02	12475	17047	14.6	85.4	2.8
**1200 ALD cycles**	1100	6.07	67244	120288	35.9	64.1	3.0
	1800	16.02	9633	48296	16.6	83.4	3.2
**2000 ALD cycles**	6000	61.88	18.9	432.9	4.2	95.8	2.9

**Figure 6:**
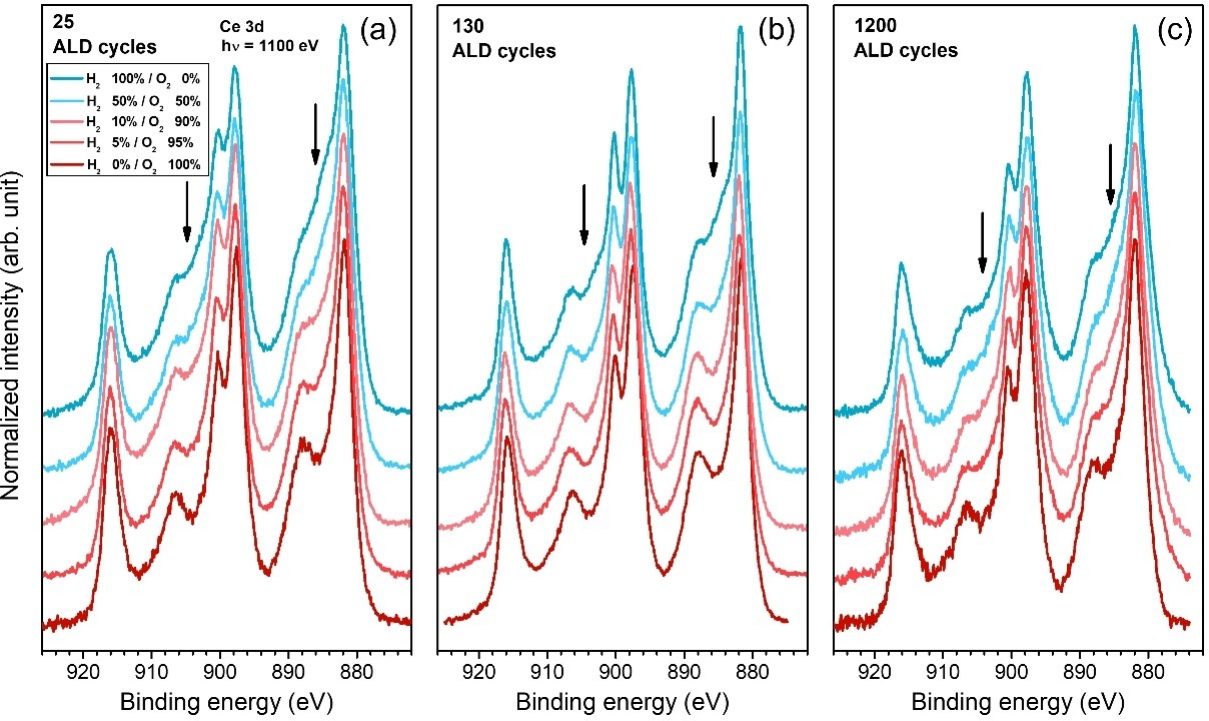
Ce 3d PES spectra for ALD‐ceria films with (a) 25, (b) 130, and (c) 1200 cycles taken at room temperature under a mixture of O_2_ and H_2_, where the P(H_2_)/P(O_2_) ratio is varied from 100/0 % (top) to 95/5 %, 90/10 %, 50/50 % and 0/100 % H_2_/O_2_ (bottom). The photon energy was fixed at 1100 eV.

**Figure 7:**
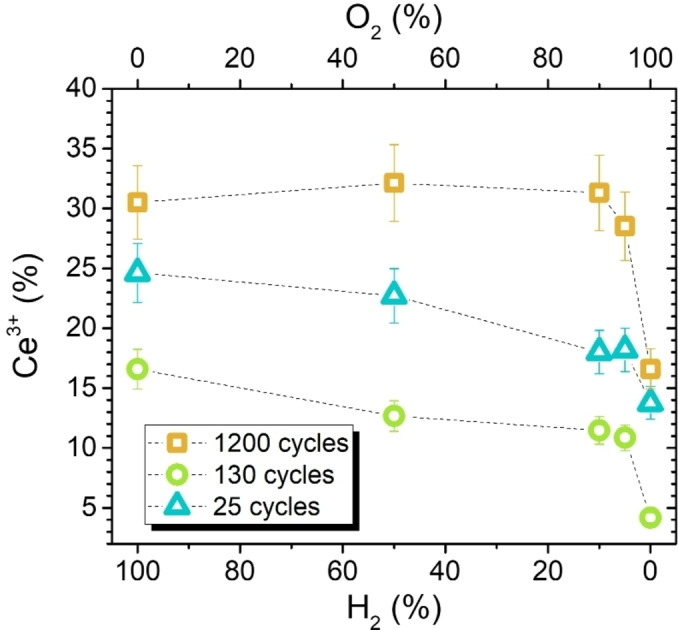
Percentage of Ce^3+^ states within 25‐CeOx (triangles), 130‐CeOx (circles), and 1200‐CeOx (squares) layers as a function of H_2_ (bottom x‐axis) and O_2_ (top x‐axis) content in the H_2_/O_2_ mixture at room temperature. The layer composition was estimated from the Ce 3d spectra shown in Figure 6.

After gaining insights into the reduction process of ALD‐ceria films in a pure H_2_ environment, we now show the results obtained when the samples are exposed to a set of O_2_/H_2_ mixtures at RT. The followed strategy consisted of starting with pure H_2_ at 10^−4^ mbar and gradually diluting it by increasing the O_2_ flow. In line with the work of Suzuki et al.^[34]^ and as discussed previously for the results shown in Figure 2 regarding the exposure to different partial pressures, this methodology should not affect the obtained reduction, as the latter will only depend on the ratio between partial pressures and not on the absolute values. Regardless of their thickness, all three ALD‐ceria samples are reduced when the total concentration of H_2_ increases, as shown by the extra Ce_2_O_3_‐related shoulders appearing at ∼884 and ∼904 eV Ce 3d spectra (see Figure 6 and the arrows therein). It is worth noting that the reduction/oxidation process at RT is reversible, reproducing the same results for multiple cycles. In detail, Figure 7 shows the concentration of Ce^3+^ states extracted from the XPS fitting of the Ce 3d spectra shown in Figure 6. Interestingly, all samples show a similar behavior, with a gradual reduction for low traces of H_2_ (<10 %) that starts saturating for higher P(H_2_)/P(O_2_) ratios. Besides, the difference between the achieved total reduction and oxidation, in terms of Ce^4+^↔Ce^3+^ conversion, is comparable in the three cases and amounts to about ten percent. Despite these similarities, some important differences depend on the deposited CeO_x_ thicknesses, probably due to interface effects. First, the total oxidation achieved by a 100 % O_2_ environment differs. In the case of the ultrathin 0.5 nm deposit (25‐CeO_x_), the higher amount of Ce^3+^ states compared to the 2.6 nm (130‐CeO_x_) film is due to the higher amount of Ce^3+^ states at the interface.^[49]^ Second, the difference between the intermediate (130‐CeOx) and 24 nm thick samples (1200‐CeOx) is even more surprising. Although the interface effect should not be so strong here, as confirmed by the high degree of oxidation achieved by the 2.6 nm thick sample, the thicker sample exhibits a higher degree of reduction for all P(H_2_)/P(O_2_) ratios. Since the interface effect can be ruled out, the most plausible explanation seems to involve an intrinsic change in the ceria matrix depending on the number of ALD cycles. Third, the thicker deposit maintains the same surface composition until the O_2_ concentration is higher than 90 %, in contrast to thinner films where the oxidation/reduction is gradual, indicating that the 24 nm thick film is less prone to oxidation and presents a higher amount of intrinsic Ce^3+^ states. Thus, thinner layers show a less stable structure that allows more pronounced changes depending on the environmental conditions.

### Reduction Reversibility after Post‐Deposition Annealing at 250 °C

The intrinsic differences between the 130‐CeO_x_ and 1200‐CeO_x_ deposits have been studied more thoroughly. As extensively discussed in our previous work,^[49]^ the reaction mechanism of the ALD process presents two different regimes depending on the surface on which the organometallic precursor reacts: the substrate or the ceria deposit. The transition between these regimes is extended over several hundreds of ALD cycles, i. e., several nanometers, affecting ceria composition and density of defects, as shown by XPS and Raman measurements. It was then hypothesized that, as the increase in the number of ALD cycles would imply that the ceria deposit was kept longer at 250 °C (sample temperature during the ALD process), this would eventually act equivalent to a post‐deposition annealing treatment in terms of healing the intrinsic defects of the ALD process.^[71]^ In other words, thinner ALD‐ceria deposits would present more defects than thicker samples kept at 250 °C for a longer time, enhancing their reducibility.

**Figure 8:**
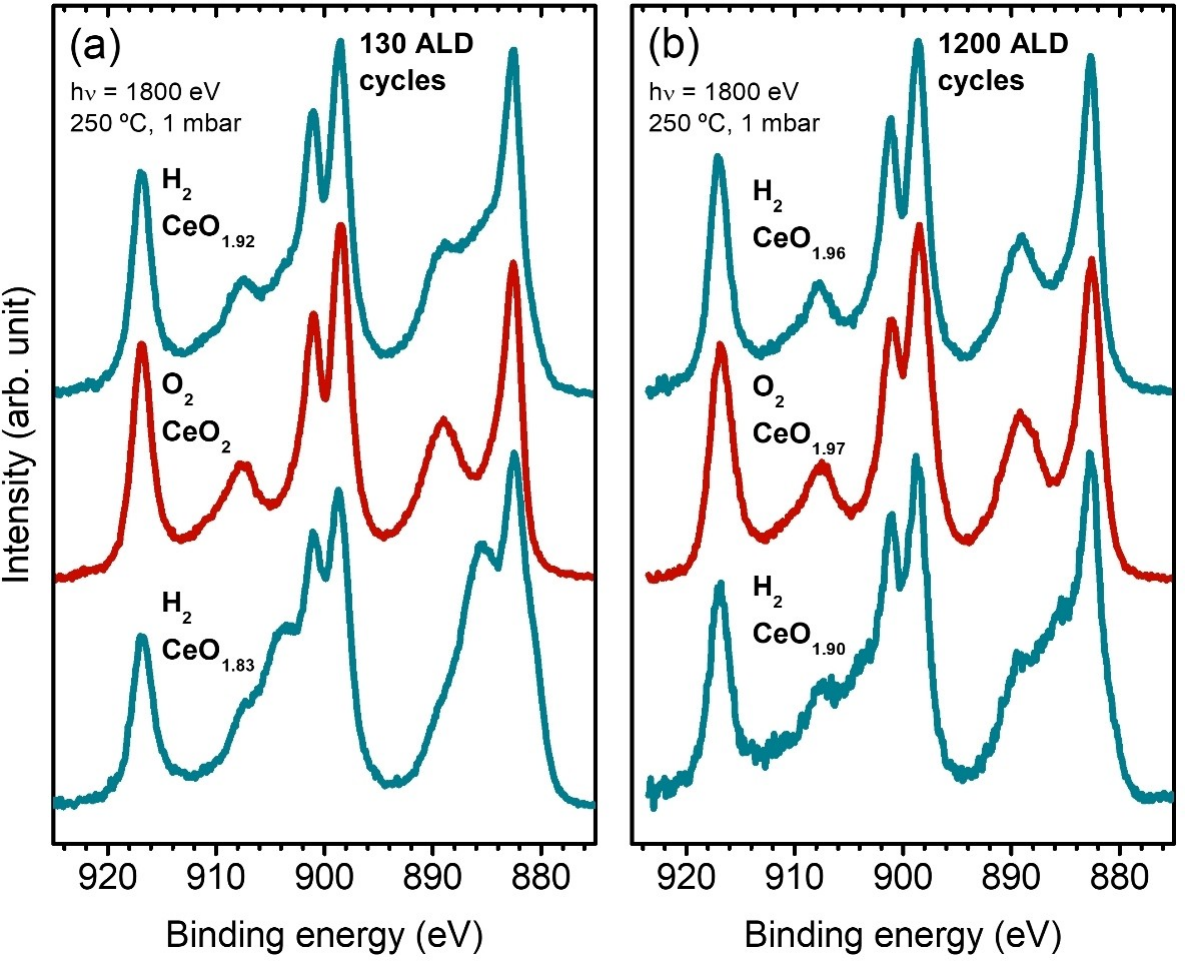
From bottom to top, Ce 3d PES spectra of 130‐CeO_x_ (a), and 1200‐CeO_x_ (b) samples subsequently taken at 250 °C under 1 mbar of H_2_, O_2_, and H_2_, respectively. The measurements were performed after 1 hour under each specific condition to allow the system to reach saturation. The composition (CeO_x_) estimated from the spectra fitting is shown for each stage. The photon energy was set at 1800 eV.

In order to prove that the ceria reducibility strongly depends on these initial states and that their disappearance at elevated heat treatment leads to a lower reactivity, we used NAP‐XPS to monitor a post‐annealing process at 250 °C, i. e., at the same temperature as the ALD growth takes place. This treatment consisted of three steps at a total pressure of 1 mbar (while keeping the temperature fixed at 250 °C throughout): (1) sample reduction under H_2_, (2) subsequently back‐oxidizing under an O_2_ environment, and (3) finally reducing the ceria again with H_2_ to investigate the reversibility of the process. Figure 8 shows three representative Ce 3d spectra of the thinner 130‐CeO_x_ (a) and thicker 1200‐CeO_x_ (b) deposits after saturation was reached in each step (after approximately one hour under each condition). Both samples are partially reduced during the first exposure to H_2_, slightly more in the case of the thinner film (CeO_1.83_ compared to CeO_1.90_), which supports the previous discussion regarding the role of the initial intrinsic defects within the film (as opposed to the trapped defects at the interface). It is worth noting that using a lower photon energy, i. e., lower kinetic energies and thus smaller IMFP values, yields a higher apparent reduction, especially for the thinner film, indicating that the ceria films present a Ce^3+^ concentration gradient in the saturated state.[^62^, ^66^] The exposure to O_2_ results in a fast, almost complete oxidation of both films. However, the second exposure to H_2_ leads to a significantly lower reduction of ceria. Compared to the 24 nm thick film, the thinner 2.6 nm deposit is significantly more affected by this loss of reducibility, as the relative change in the amount of Ce^3+^ states generated between both reducing treatments is almost double, which supports our interpretation that annealing treatments, whether during ALD‐process or after it, heal the intrinsic ceria defects from the ALD process and hence decrease the reactivity of the oxide.

**Figure 9:**
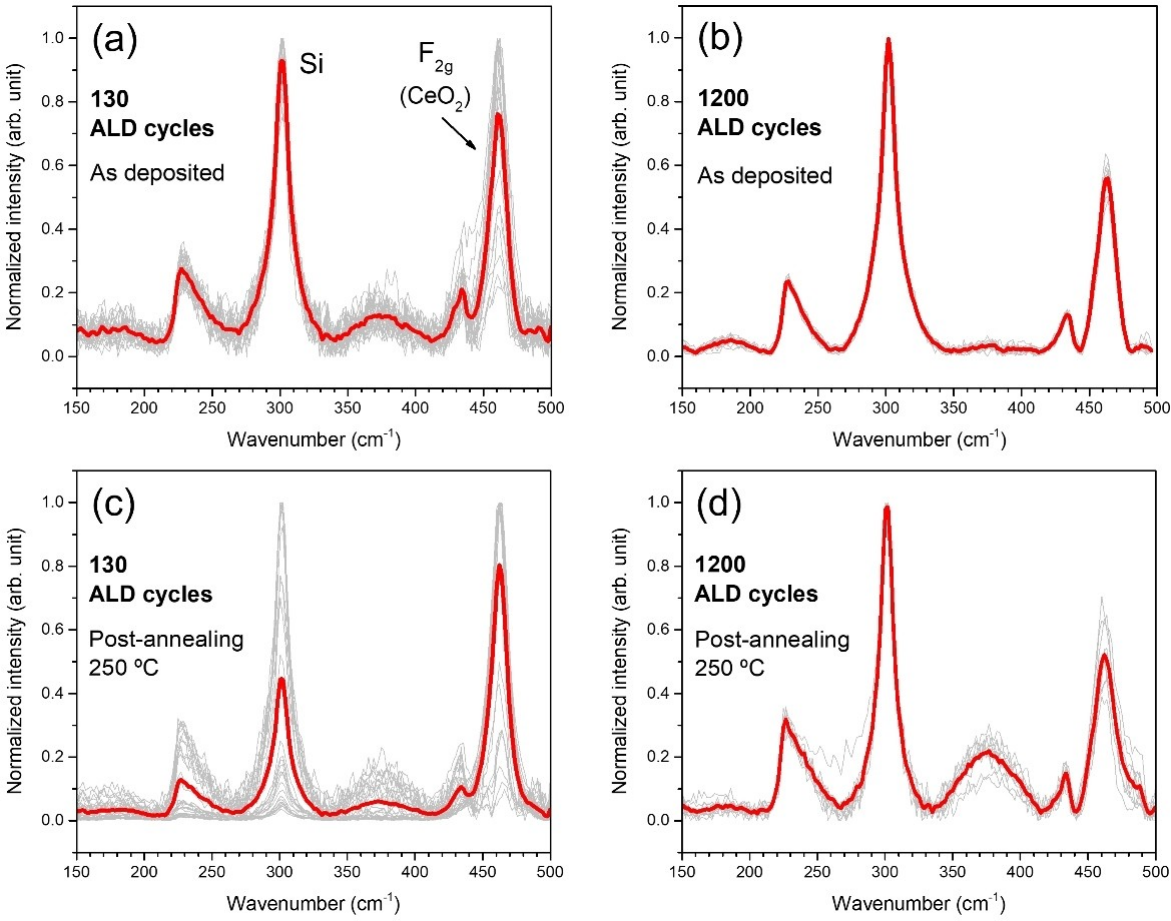
Raman spectra of ALD films 130‐CeO_x_ and 1200‐CeO_x_ as deposited, (a) and (b), and after the post‐annealing process at 250 °C, (c) and (d), respectively. The post‐deposition annealing process corresponds to that indicated in Figure 8. The red spectra are the average of several individual spectra (light grey) taken at different positions of the layer surface.

The same ALD‐ceria films, 130‐CeO_x_ and 1200‐CeO_x_, have been characterized by *ex‐situ* μ‐Raman spectroscopy before (a, b) and after (c, d) the above‐described three‐step post‐annealing process, respectively. As shown in Figure 9, multiple measurements were performed at different positions of the layer surface (light grey), showing the average spectrum in red. The thinner sample exhibits a large scatter due to an incomplete coalescence of the film at this stage, resulting in an inhomogeneous film morphology consisting of micrometric flakes.^[49]^ The most remarkable result comes from comparing the intensity of the F_2g_ band at ~460 cm^−1^ with the Si signal from the substrate.^[72]^ Whereas the 1200‐CeO_x_ sample preserves almost the same intensity ratio upon annealing, the 130‐CeO_x_ deposit presents a substantial change, increasing the F_2g_ band intensity, which would be a fingerprint of ceria crystallization induced by post‐annealing. The absence of a significant change in the data of the thicker layer is likely due to the mild annealing conditions applied. For example, Coll and co‐authors reported epitaxial ALD ceria deposits after sample annealing at much higher temperatures, between 700 and 900 °C.^[71]^ A more detailed Raman analysis would require a discussion of the ceria bands related to surface phonon modes appearing at 270 and 315 cm^−1^ for crystal sizes below 10 nm,^[73]^ as well as the broad ceria defects D‐band at 550–600 cm^−1^.^[72]^ Unfortunately, these bands overlap with the intense Raman signals of the Si substrate at 302 and 520 cm^−1^, preventing a comprehensive analysis of the changes in the ceria matrix when grown on SiO_2_ substrates.

**Figure 10:**
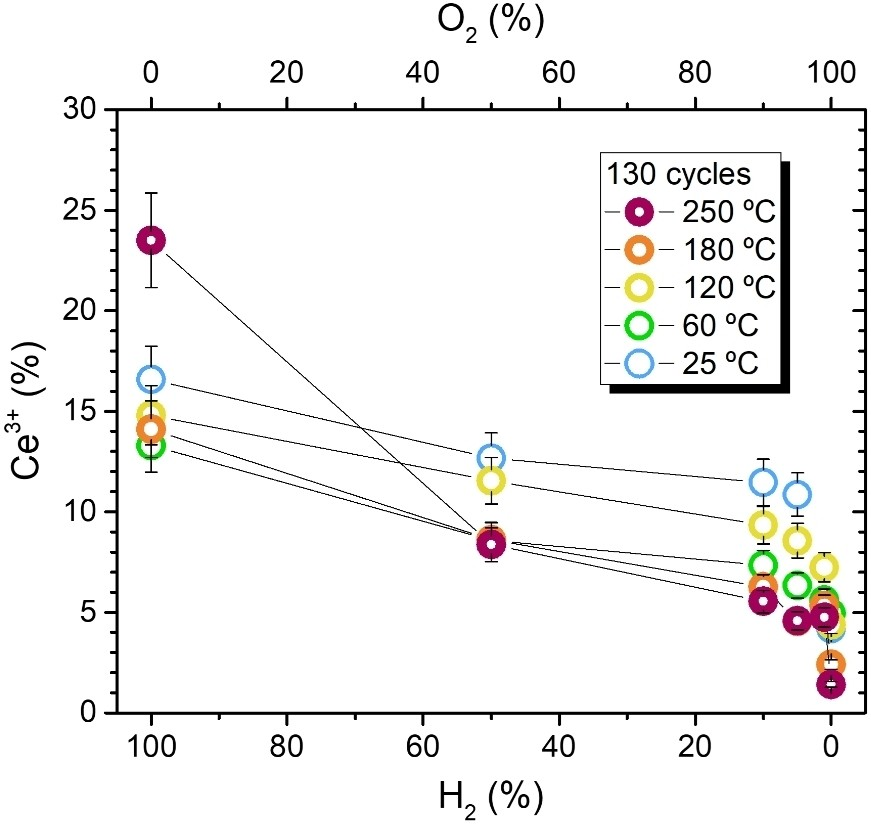
Percentage of Ce^3+^ states for the 130‐CeO_x_ layer as a function of H_2_ (bottom x‐axis) and O_2_ (top x‐axis) content in the H_2_/O_2_ mixture at temperatures ranging from 25 °C to 250 °C).

It is worth noting that the conversion between Ce^4+^ to Ce^3+^ states, although slightly weakened due to the recrystallization process, continues during exposure to low H_2_ concentrations within an oxidizing atmosphere at higher temperatures. Figure 10 displays the Ce^4+^/Ce^3+^ change of an equivalent 130‐CeO_x_ film at increasing temperatures up to 250 °C when exposed to the same H_2_/O_2_ mixtures as depicted in Figure 7. While H_2_ concentrations above 50 % and high temperatures (>200 °C) promote an increased reduction likely due to the formation of oxygen vacancies at the surface followed by their diffusion, at lower concentrations, the same step‐like behavior as at room temperature is observed, yet resulting in a slightly lower Ce^3+^ concentration due to the induced recrystallization. Therefore, although the partial healing of the ALD‐intrinsic defects will decrease the potential reducibility of the ceria in terms of heterolytic activation of the hydrogen molecule, higher temperatures allow overcoming the energy barrier associated with the strong bond of the hydroxyl group, thus releasing a H_2_O molecule and leaving and oxygen vacancy at the surface (see equation 2), especially when P(H_2_) is higher than P(O_2_).

### Sensing Response Towards H_2_

In the previous sections, we have provided clear evidence that ALD‐ceria deposits of different thicknesses are partially reduced after exposure to pure H_2_ atmosphere at room temperature. In O_2_ and H_2_ gas mixtures, the reduction of the ALD ceria surface depends on the P(H_2_)/P(O_2_) ratio, slightly varying with H_2_ concentration, but especially in the range from 0 to 10 %. NAP‐XPS measurements show that the H_2_ molecules easily dissociate, leading to hydroxyl species through a charge transfer that reduces the surface Ce^4+^ cations to Ce^3+^. As thoroughly discussed in the literature, the release of these electrons into the conduction band would increase the conductivity of the ALD‐ceria film and thus enable a sensor response based on a resistance decrease.^[2]^

**Figure 11:**
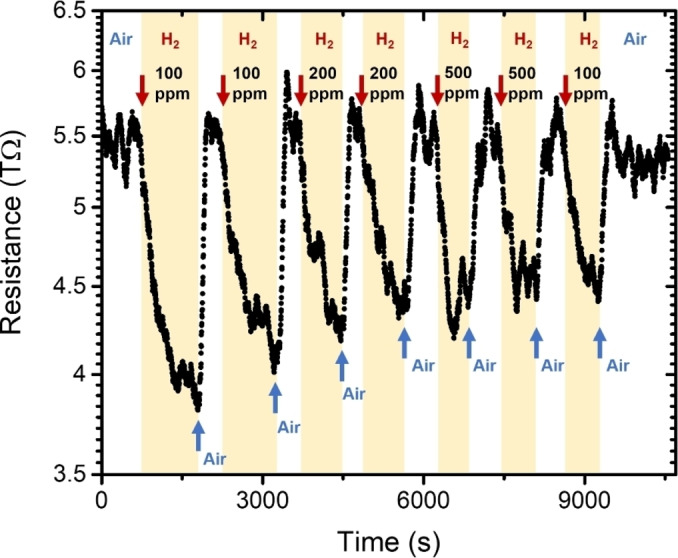
Changes in electrical resistance of sensor test structure with an ALD‐CeO_x_ (20 nm) active layer after exposure to consecutive H_2_ doses (in the range of 100–500 ppm diluted in Ar) at an operating temperature of 180 °C. The recovery was performed with air.

Figure 11 shows the change in the electrical resistance of a sensor test structure comprising a 20 nm thick bare ceria deposit towards diluted H_2_ in an Ar flow, proving a sensor response ($\frac{R_{air}}{R_{H_{2}}}$ ) of about 1.4 for relatively low doses and operating temperatures. Interestingly, 180 °C constitutes the lowest temperature at which the ALD‐ceria layer shows a readable electrical response. Considering the trends of Figure 10, such operating conditions seem a good compromise between overcoming the energy barrier for oxygen vacancy formation and diffusion towards the subsurface region and limited healing of intrinsic ALD defects. Taking into account the previous discussion about growth conditions, although thinner films would likely show lower minimum operating temperatures due to a higher reactivity, their higher resistance makes it difficult to measure any response with our experimental setup. Importantly, ALD‐ceria layers show a sensing response to H_2_ in a diluted environment without the need to decorate the surface with a noble metal to promote a significant H_2_ dissociation.^[35]^ Compared to ceria with higher crystallinity,[^34^, ^62^, ^63^, ^66^] the intrinsic structural defects of the ALD deposit possibly play a fundamental role by creating electronic defect states that lower the energy barriers and allow a significant reduction of ceria even at room temperature.

We note that the high resistance of the ALD active layer is related to its low thickness and lack of device optimization (e. g., the specific design of the interdigitated electrodes depending on film thickness and resistivity). Additionally, structural defects at the interface with the substrate will likely act as charge traps and thus increase the sheet resistance of ALD‐ceria films, consequently increasing the overall resistivity and limiting the sensor response at lower operating temperatures.

Different strategies can be proposed to increase the ALD‐ceria response while lowering its overall resistance. On the one hand, traditional surface decoration by noble metal nanoparticles, such as Pd, will likely increase the sensor response by helping dissociate the H_2_ molecule, thus facilitating ceria reduction beyond the topmost layer at low temperatures. On the other hand, one way to overcome the high resistance of ALD‐ceria layers is to combine highly reactive ultrathin ALD‐ceria with other, more conductive metal oxides within well‐defined sensor heterostructures that would directly benefit from the H_2_‐ceria charge transfer. This approach, which does not require the use of noble metals, would be similar to, but more controllable than, the use of In_2_O_3_‐ceria^[38]^ and SnO_2_‐ceria^[39]^ solid solutions for H_2_ sensing.

## Conclusions

Reducing ceria at room temperature without using noble metals constitutes a promising result for applications in multiple fields, such as CO_2_ conversion in catalysis or H_2_ sensing, both cornerstone examples that need to be addressed within the imminent transition towards a green and renewable energy system. Based on an extensive combination of *in‐situ* and *ex‐situ* experimental techniques, we have demonstrated that ultrathin atomic layer deposited ceria with thicknesses between 0.5 and 24 nm become significantly reduced at room temperature when exposed to an H_2_ atmosphere regardless of the absolute pressure. In H_2_/O_2_ mixtures, ALD‐ceria can gradually be reduced as a function of H_2_ concentration, especially for mixtures with H_2_ concentrations below 10 %. Although this reduction is limited to the surface region at room temperature, the hydroxylation of the ceria surface induces a charge transfer towards the ceria film that allows the reduction of Ce^4+^ cations to Ce^3+^, thus replicating the expected sensing mechanism for metal oxides at low temperatures and without the use of any noble metal to promote a significant H_2_ dissociation. The sensing response of bare ALD‐ceria films towards diluted H_2_ doses has successfully been demonstrated in sensor test structures.

The intrinsic defects of the ALD deposit seem to play a crucial role in this reduction at room temperature, showing less reactivity when subjected to a post‐deposition annealing process capable of healing these intrinsic defects. The long times required for growing thick layers by ALD and the relatively high temperatures of the process (250 °C) might explain why thicker films have a more stable structure and thus lower reactivity compared to ultrathin layers (~2.5 nm). Nevertheless, we have found that recrystallized ALD‐ceria layers are still chemically active; also, moderate operation temperatures (below 200 °C) represent a good compromise between a reasonably low defect‐healing rate and temperature‐activated oxygen vacancy formation. These promising results call for further investigations combining ALD‐ceria with more conductive metal oxides, such as SnO_2_ or In_2_O_3_, taking advantage of more facile charge transfer processes at the interface and thus modifying the depletion layer created at the heterojunction. Moreover, future works should thoroughly focus on the sensing properties of highly defective ALD‐ceria active layers integrated into tailored sensor structures, determining and optimizing sensor properties such as response and recovery times, cross‐sensitivity with other reducing agents, and electrical response under air and humidity.

## Conflict of Interests

The authors declare no conflict of interest.

## Data Availability

The data that support the findings of this study are available from the corresponding author upon reasonable request.

## References

[1] T. Hübert , L. Boon-Brett , G. Black , U. Banach , Sens. Actuators B 2011, 157 (2), 329–352. 10.1016/j.snb.2011.04.070.

[2] C. Wang , L. Yin , L. Zhang , D. Xiang , R. Gao , Sensors 2010, 10 (3), 2088–2106. 10.3390/s100302088. 22294916 PMC3264469

[3] S. Phanichphant , Procedia Eng. 2014, 87, 795–802. 10.1016/j.proeng.2014.11.677.

[4] J. M. Walker , S. A. Akbar , P. A. Morris , Sens. Actuators B 2019, 286, 624–640. 10.1016/j.snb.2019.01.049.

[5] M. V. Nikolic , V. Milovanovic , Z. Z. Vasiljevic , Z. Stamenkovic , Sensors 2020, 20 (22), 6694. 10.3390/s20226694. 33238459 PMC7700484

[6] A. Katsuki , K. Fukui , Sens. Actuators B 1998, 52 (1–2), 30–37. 10.1016/S0925-4005(98)00252-4.

[7] S. Lu , Y. Zhang , J. Liu , H.-Y. Li , Z. Hu , X. Luo , N. Gao , B. Zhang , J. Jiang , A. Zhong , J. Luo , H. Liu , Sens. Actuators B 2021, 345, 130334. 10.1016/j.snb.2021.130334.

[8] T. Anderson , F. Ren , S. Pearton , B. S. Kang , H.-T. Wang , C.-Y. Chang , J. Lin , Sensors 2009, 9 (6), 4669–4694. 10.3390/s90604669. 22408548 PMC3291933

[9] N. H. Al-Hardan , M. J. Abdullah , A. A. Aziz , Int. J. Hydrogen Energy 2010, 35 (9), 4428–4434. 10.1016/j.ijhydene.2010.02.006.

[10] B. Mondal , B. Basumatari , J. Das , C. Roychaudhury , H. Saha , N. Mukherjee , Sens. Actuators B 2014, 194, 389–396. 10.1016/j.snb.2013.12.093.

[11] H. Gu , Z. Wang , Y. Hu , Sensors 2012, 12 (5), 5517–5550. 10.3390/s120505517. 22778599 PMC3386698

[12] G. Zhang , M. Liu , Sens. Actuators B 2000, 69 (1–2), 144–152. 10.1016/S0925-4005(00)00528-1.

[13] G. Korotcenkov , Sens. Actuators B 2005, 107 (1), 209–232. 10.1016/j.snb.2004.10.006.

[14] P. Yang , D. Zhao , D. I. Margolese , B. F. Chmelka , G. D. Stucky , Nature 1998, 396 (6707), 152–155. 10.1038/24132.

[15] U. Ciesla , F. Schüth , Microporous Mesoporous Mater. 1999, 27 (2–3), 131–149. 10.1016/S1387-1811(98)00249-2.

[16] O. Lupan , G. Chai , L. Chow , Microelectron. Eng. 2008, 85 (11), 2220–2225. 10.1016/j.mee.2008.06.021.

[17] O. Lupan , V. V. Ursaki , G. Chai , L. Chow , G. A. Emelchenko , I. M. Tiginyanu , A. N. Gruzintsev , A. N. Redkin , Sens. Actuators B 2010, 144 (1), 56–66. 10.1016/j.snb.2009.10.038.

[18] J.-H. Lee , Sens. Actuators B 2009, 140 (1), 319–336. 10.1016/j.snb.2009.04.026.

[19] X.-T. Yin , L. Tao , J. Alloys Compd. 2017, 727, 254–259. 10.1016/j.jallcom.2017.08.122.

[20] O. Lupan , V. Postica , N. Wolff , J. Su , F. Labat , I. Ciofini , H. Cavers , R. Adelung , O. Polonskyi , F. Faupel , L. Kienle , B. Viana , T. Pauporté , ACS Appl. Mater. Interfaces 2019, 11 (35), 32115–32126. 10.1021/acsami.9b08598. 31385698

[21] X. T. Yin , W. D. Zhou , J. Li , Q. Wang , F. Y. Wu , D. Dastan , D. Wang , H. Garmestani , X. M. Wang , Ş. Ţălu , J. Alloys Compd. 2019, 805, 229–236. 10.1016/j.jallcom.2019.07.081.

[22] N. X. Thai , N. Van Duy , N. Van Toan , C. M. Hung , N. Van Hieu , N. D. Hoa , Int. J. Hydrogen Energy 2020, 45 (3), 2418–2428. 10.1016/j.ijhydene.2019.11.072.

[23] A. Mirzaei , H. R. Yousefi , F. Falsafi , M. Bonyani , J.-H. Lee , J.-H. Kim , H. W. Kim , S. S. Kim , Int. J. Hydrogen Energy 2019, 44 (36), 20552–20571. 10.1016/j.ijhydene.2019.05.180.

[24] Y. Luo , C. Zhang , B. Zheng , X. Geng , M. Debliquy , Int. J. Hydrogen Energy 2017, 42 (31), 20386–20397. 10.1016/j.ijhydene.2017.06.066.

[25] D. R. Mullins , Surf. Sci. Rep. 2015, 70 (1), 42–85. 10.1016/j.surfrep.2014.12.001.

[26] X. Wang , J. Wang , Y. Sun , K. Li , T. Shang , Y. Wan , Front. Chem. 2022, 10. 10.3389/fchem.2022.1089708 PMC9772620 36569964

[27] H.-J. Beie , A. Gnörich , Sens. Actuators B 1991, 4 (3–4), 393–399. 10.1016/0925-4005(91)80141-6.

[28] N. Izu , W. Shin , N. Murayama , S. Kanzaki , Sens. Actuators B 2002, 87 (1), 95–98. 10.1016/S0925-4005(02)00224-1.

[29] D. Barreca , A. Gasparotto , C. Maccato , C. Maragno , E. Tondello , E. Comini , G. Sberveglieri , Nanotechnology 2007, 18 (12), 125502. 10.1088/0957-4484/18/12/125502.

[30] A. A. Aboud , H. Al-Kelesh , W. M. A. E. Rouby , A. A. Farghali , A. Hamdedein , M. H. Khedr , J. Mater. Res. Technol. 2018, 7 (1), 14–20. 10.1016/j.jmrt.2017.03.003.

[31] D. K. Subbiah , A. J. Kulandaisamy , R. B. George , P. Shankar , G. K. Mani , K. Jayanth Babu , J. B. B. Rayappan , J. Alloys Compd. 2018, 753, 771–780. 10.1016/j.jallcom.2018.04.248.

[32] E. R. López-Mena , C. R. Michel , A. H. Martínez-Preciado , A. Elías-Zuñiga , Nanoscale Res. Lett. 2017, 12 (1), 169. 10.1186/s11671-017-1951-x. 28274087 PMC5339090

[33] D. Majumder , S. Roy , ACS Omega 2018, 3 (4), 4433–4440. 10.1021/acsomega.8b00146. 31458670 PMC6641583

[34] K. Suzuki , H. Miyazaki , Y. Yuzuriha , Y. Maru , N. Izu , Sens. Actuators B 2017, 250, 617–622. 10.1016/j.snb.2017.05.008.

[35] Y. Song , X. Meng , M. Bi , W. Gao , Sens. Actuators B 2023, 375, 132957. 10.1016/j.snb.2022.132957.

[36] H. Hashtroudi , A. Yu , S. Juodkazis , M. Shafiei , Nanomaterials 2022, 12 (10), 1628. 10.3390/nano12101628. 35630850 PMC9147198

[37] D. Baier , T. Priamushko , C. Weinberger , F. Kleitz , M. Tiemann , ACS Sens. 2023, 8 (4), 1616–1623. 10.1021/acssensors.2c02739. 37017638

[38] J. Hu , Y. Sun , Y. Xue , M. Zhang , P. Li , K. Lian , S. Zhuiykov , W. Zhang , Y. Chen , Sens. Actuators B 2018, 257, 124–135. 10.1016/j.snb.2017.10.139.

[39] D. E. Motaung , H. G. Mhlongo , P. R. Makgwane , B. P. Dhonge , F. R. Cummings , H. C. Swart , S. S. Ray , Sens. Actuators B 2018, 254, 984–995. 10.1016/j.snb.2017.07.093.

[40] V. Cremers , R. L. Puurunen , J. Dendooven , Appl. Phys. Rev. 2019, 6 (2), 021302. 10.1063/1.5060967.

[41] N. E. Richey , C. de Paula , S. F. Bent , J. Chem. Phys. 2020, 152 (4), 040902. 10.1063/1.5133390. 32007080

[42] P. O. Oviroh , R. Akbarzadeh , D. Pan , R. A. M. Coetzee , T.-C. Jen , Sci. Technol. Adv. Mater. 2019, 20 (1), 465–496. 10.1080/14686996.2019.1599694. 31164953 PMC6534251

[43] M. Coll , M. Napari , APL Mater. 2019, 7 (11), 110901. 10.1063/1.5113656.

[44] V. Yu , J. Struct. Chem. 2022, 63 (7), 1019–1050. 10.1134/S0022476622070022.

[45] J. Strand , A. L. Shluger , Adv. Sci. 2024, 11 (8), 2306243. 10.1002/advs.202306243. PMC10885675 38148443

[46] C. Morales , A. Mahmoodinezhad , R. Tschammer , J. Kosto , C. Alvarado Chavarin , M. A. Schubert , C. Wenger , K. Henkel , J. I. Flege , Inorganics 2023, 11 (12), 477. 10.3390/inorganics11120477.

[47] T. V. Ivanova , J. Toivonen , P. S. Maydannik , T. Kääriäinen , M. Sillanpää , T. Homola , D. C. Cameron , J. Vac. Sci. Technol. A: Vacuum, Surfaces, Films 2016, 34 (3), 031506. 10.1116/1.4944589.

[48] J. Päiväsaari , M. Putkonen , L. Niinistö , J. Mater. Chem. 2002, 12 (6), 1828–1832. 10.1039/b108333c.

[49] C. Morales , M. Gertig , M. Kot , C. Alvarado Chavarin , M. A. Schubert , M. H. Zoellner , C. Wenger , K. Henkel , J. I. Flege , Adv. Mater. Interfaces 2024. 10.1002/admi.202400537.

[50] T. Gross , M. Ramm , H. Sonntag , W. Unger , H. M. Weijers , E. H. Adem , Surf. Interface Anal. 1992, 18 (1), 59–64. 10.1002/sia.740180110.

[51] V. Pérez-Dieste , L. Aballe , S. Ferrer , J. Nicolàs , C. Escudero , A. Milán , E. Pellegrin , J. Phys. Conf. Ser. 2013, 425 (7), 072023. 10.1088/1742-6596/425/7/072023.

[52] M. Favaro , P. C. J. Clark , M. J. Sear , M. Johansson , S. Maehl , R. Krol , D. E. van de Starr , Surf. Sci. 2021, 713, 121903. 10.1016/j.susc.2021.121903.

[53] E. Paparazzo , J. Phys. Condens. Matter 2018, 30 (34), 343003. 10.1088/1361-648X/aad248. 29988022

[54] M. Romeo , K. Bak , J. El Fallah , F. Le Normand , L. Hilaire , Surf. Interface Anal. 1993, 20 (6), 508–512. 10.1002/sia.740200604.

[55] T. Skála , F. Šutara , K. C. Prince , V. Matolín , J. Electron Spectrosc. Relat. Phenom. 2009, 169 (1), 20–25. 10.1016/j.elspec.2008.10.003.

[56] S. Tanuma , C. J. Powell , D. R. Penn , Surf. Interface Anal. 1994, 21 (3), 165–176. 10.1002/sia.740210302.

[57] S. D. Elliott , Semicond. Sci. Technol. 2012, 27 (7), 074008. 10.1088/0268-1242/27/7/074008.

[58] F. Dvořák , O. Stetsovych , M. Steger , E. Cherradi , I. Matolínová , N. Tsud , M. Škoda , T. Skála , J. Mysliveček , V. Matolín , J. Phys. Chem. C 2011, 115 (15), 7496–7503. 10.1021/jp1121646.

[59] D. Zhang , X. Du , L. Shi , R. Gao , Dalton Trans. 2012, 41 (48), 14455. 10.1039/c2dt31759a. 23027607

[60] P. Luches , S. Valeri , Materials 2015, 8 (9), 5818–5833. 10.3390/ma8095278. 28793536 PMC5512658

[61] J. I. Flege , B. Kaemena , J. Höcker , F. Bertram , J. Wollschläger , T. Schmidt , J. Falta , Appl. Phys. Lett. 2014, 104 (13), 131604. 10.1063/1.4870585.

[62] J. Höcker , T. O. Menteş , A. Sala , A. Locatelli , T. Schmidt , J. Falta , S. D. Senanayake , J. I. Flege , Adv. Mater. Interfaces 2015, 2 (18), 1500314. 10.1002/admi.201500314.

[63] J. Höcker , T. Duchoň , K. Veltruská , V. Matolín , J. Falta , S. D. Senanayake , J. I. Flege , J. Phys. Chem. C 2016, 120 (9), 4895–4901. 10.1021/acs.jpcc.5b11066.

[64] J. Xu , S. H. Overbury , J. Catal. 2004, 222 (1), 167–173. 10.1016/j.jcat.2003.10.011.

[65] D. Fernández-Torre , J. Carrasco , M. V. Ganduglia-Pirovano , R. Pérez , J. Chem. Phys. 2014, 141 (1), 014703. 10.1063/1.4885546. 25005299

[66] T. Duchoň , J. Hackl , D. N. Mueller , J. Kullgren , D. Du , S. D. Senanayake , C. Mouls , D. M. Gottlob , M. I. Khan , S. Cramm , K. Veltruská , V. Matolín , S. Nemšák , C. M. Schneider , J. Mater. Chem. A 2020, 8 (11), 5501–5507. 10.1039/C9TA11784A.

[67] F. A. Kröger, H. J. Vink, *Solid State Phys*.; Elsevier, **1956**; Vol. 3, pp 307–435. 10.1016/S0081-1947(08)60135-6.

[68] Z. A. Feng , F. El Gabaly , X. Ye , Z.-X. Shen , W. C. Chueh , Nat. Commun. 2014, 5 (1), 4374. 10.1038/ncomms5374. 25007038

[69] A. Allahgholi , J. I. Flege , S. Thieß , W. Drube , J. Falta , ChemPhysChem 2015, 16 (5), 1083–1091. 10.1002/cphc.201402729. 25703923

[70] M. Capdevila-Cortada , N. López , Nat. Mater. 2017, 16 (3), 328–334. 10.1038/nmat4804. 27869825

[71] M. Coll , J. Gazquez , A. Palau , M. Varela , X. Obradors , T. Puig , Chem. Mater. 2012, 24 (19), 3732–3737. 10.1021/cm301864c.

[72] S. Loridant , Catal. Today 2021, 373, 98–111. 10.1016/j.cattod.2020.03.044.

[73] S. Wang , W. Wang , J. Zuo , Y. Qian , Mater. Chem. Phys. 2001, 68 (1–3), 246–248. 10.1016/S0254-0584(00)00357-6.

